# Alum‐adjuvanted allergoids induce functional IgE‐blocking antibodies

**DOI:** 10.1111/cea.13120

**Published:** 2018-03-23

**Authors:** M. Reithofer, S. L. Böll, C. Kitzmüller, F. Horak, M. Sotoudeh, B. Bohle, B. Jahn‐Schmid

**Affiliations:** ^1^ Institute of Pathophysiology and Allergy Research Medical University of Vienna Vienna Austria; ^2^ Allergiezentrum Wien West Vienna Austria

To the Editor

The only therapy that is able to modulate the cause of IgE‐mediated allergy and to attain a long‐term effect is allergen‐specific immunotherapy (AIT). In conventional subcutaneous AIT, the vaccine consists of an extract from an allergen source that contains major and minor allergens as well as non‐allergenic proteins. To reduce IgE‐mediated side‐effects caused by the injection of intact allergens, chemically modified extracts with less IgE‐binding activity, named allergoids, have been used for AIT since the 1980s. Most allergoids are obtained by treating allergen extracts with form‐ or glutaraldehyde, which react with free amino groups and form covalent inter‐ and intramolecular bonds, resulting in the formation of high‐molecular‐weight aggregates. Allergoids were intended to increase the efficacy of AIT and to reduce the frequency of injections, as the reduced IgE‐reactivity makes it possible to apply higher vaccine doses. Clinical efficacy of allergoids has been shown and reviewed in the past^1^, ^2^ and presently, they are routinely used for AIT.

In spite of their reduced IgE‐binding and allergenicity, allergoids have been shown to be immunogenic and to induce allergen‐specific IgG, especially IgG1 and IgG4.^2^ Such antibodies in sera from patients receiving subcutaneous AIT with unmodified allergens have been reported to block IgE‐activity.^3^, ^4^ It has been discussed that IgG antibodies induced by allergoids also have blocking capacity^5^, ^6^; however, their functional blocking capacity has not been demonstrated yet. We employed sera from patients allergic to grass pollen (GP) who were undergoing preseasonal AIT with an allergoid vaccine to investigate their IgE‐blocking capacity. The inhibition of IgE‐activity in patients’ sera was investigated using two different biological in vitro tests: i) inhibition of facilitated antigen binding (FAB) to the low‐affinity receptor for IgE (FcεRII, CD23) and ii) inhibition of IgE cross‐linking and activation of allogeneic basophils.

Sera and whole blood samples of ten GP‐allergic patients (3 women, 7 men; mean age: 30 years; range 19‐43 years) undergoing subcutaneous AIT with a vaccine containing a formaldehyde‐modified GP‐extract (Allergovit^®^, Allergopharma, Reinbeck, Germany; for characteristics see^2^ and Data S1) were investigated. The included patients displayed typical clinical symptoms during the GP season, positive skin‐prick tests to GP‐extract (ALK, Hørsholm, Denmark) and elevated levels of total IgE (median: 151 kU/L) and GP‐specific IgE (median: 11.5 kU/L) as shown in Table S1. The treatment comprised an initial updosing phase (7 weekly injections) and 3 injections of the maintenance dose before the grass pollen season (Figure 1A). Peripheral blood was taken before, after 4 months and after 12 months of AIT, respectively. From 5 of these patients, blood was also collected after 15 months of AIT. Basophil sensitivity to GP‐allergens was determined in whole blood ex vivo as described in the Data S1. After stimulation with different concentrations of GP‐extract, basophil activation was assessed by detection of the granular protein CD63 on the cell surface using flow cytometry which indicates basophil degranulation. 8 ng/mL and 40 ng/mL GP‐extract proved to be optimal for most individuals and induced similar basophil activation levels (Figure 1B). After 4 months of AIT, a significant decrease in basophil activation was observed which was even more distinct after 1 year of AIT resulting in a median of less than 5% of CD63^+^ basophils, even though the vaccinations were interrupted after the 3rd maintenance dose before the grass pollen season according to the treatment schedule.

**Figure 1:**
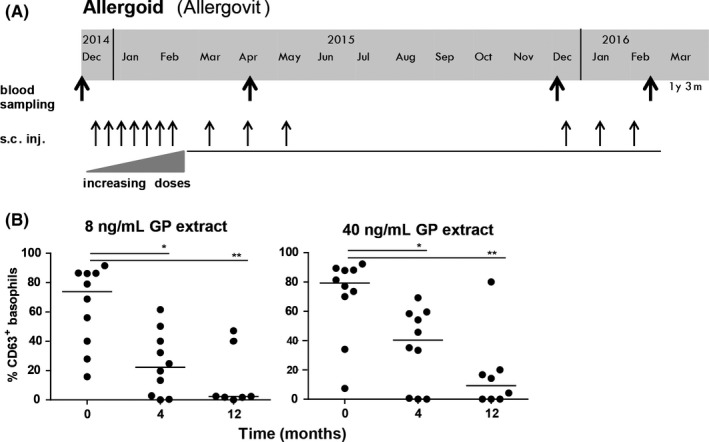
Preseasonal GP‐AIT with allergoid. The schedule of treatment and blood sampling is shown (A). Ex vivo basophil reactivity of 10 patients to GP‐extract before, after 4 and 12 months of treatment is shown (B). Grey bars represent means (Kruskal‐Wallis plus Dunn's post hoc test; *P ≤ .05; **P ≤ .01)

GP‐specific IgE, IgG, IgG1 and IgG4 antibodies induced by AIT were monitored by ELISA as described in the Data S1. While the levels of IgE against GP did not change significantly during the course of AIT (Figure 2A), allergen‐specific IgG and IgG1 levels significantly increased after 4 months of AIT in all patients. After 12 months, IgG‐ and IgG1‐levels markedly dropped down. However, after the next two preseasonal injections in the second year of AIT, the 5 patients who could be followed up for 3 more months of the vaccination, showed again increased levels of GP‐specific IgG and IgG1, which were similar to those observed after 4 months of AIT. GP‐specific IgG4 increased significantly with time. In 4 individuals, IgG4 had not yet increased after 4 months of AIT although for two of these patients, the highest concentrations of GP‐specific IgG and IgG1 were recorded at that time‐point. Unlike allergen‐specific IgG1, the levels of GP‐specific IgG4 further increased within the last 3 months in the 5 patients who were analysed after 15 months of AIT. We also determined the levels of antibodies specific for 2 representative major allergens, Phl p 1 and Phl p 5 (Figure 2B) to demonstrate that the IgG‐responses were not only directed against non‐allergenic proteins present in the allergen‐extract. These important grass pollen allergens are recognized by adult grass pollen allergic subjects with a prevalence of 90%‐100% and 60%‐80%, respectively. IgE slightly decreased during the treatment. The levels of Phl p 1‐ and Phl p 5‐specific IgG and IgG1 showed a similar course as the antibodies reacting with the whole GP‐extract, except that the initial increase and the decrease during the discontinuation period did not reach statistical significance. Similar to GP‐specific antibodies, the levels of IgG4 antibodies to individual allergens also included differential kinetics within the patients (Figure 2B,C).

**Figure 2:**
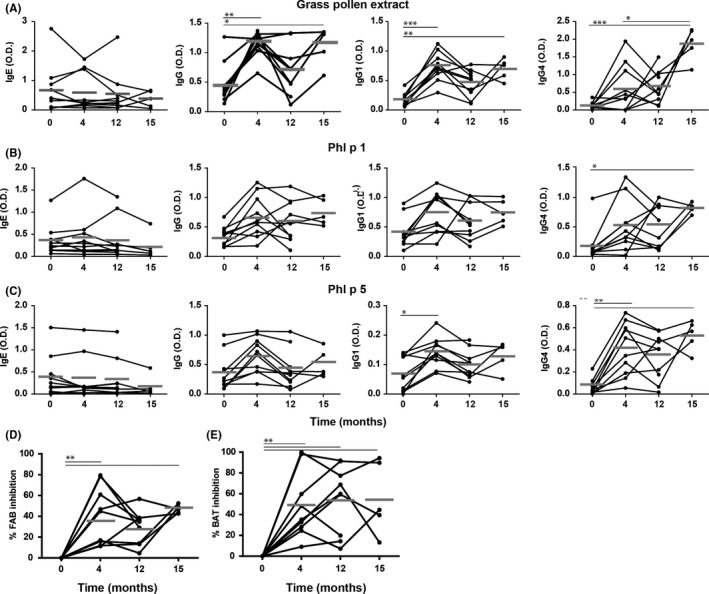
Allergen‐specific Ig‐response and IgE‐blocking capacity in patients undergoing AIT with allergoid. IgE, IgG, IgG1 and IgG4 levels were assessed by ELISA using GP‐extract (A), recombinant Phl p 1 (B) or recombinant Phl p 5 (C) for coating. Blocking capacity of sera was determined by blocking IgE‐allergen complex formation (FAB assay) (D) or by blocking the activation of basophils with GP‐extract from an allogeneic GP‐allergic individual (BAT assay) (E). Per cent inhibition as compared to pretreatment levels is shown. Grey bars represent means (Kruskal‐Wallis plus Dunn's *post hoc* test; *P ≤ .05; **P ≤ .01; ***P ≤ .001)

AIT‐induced IgE‐blocking capacity in the patients’ sera was first assessed by FAB inhibition as described in the Data S1. This assay provides information on the ability to inhibit the formation of IgE/allergen‐complexes using an allogeneic indicator serum and subsequent binding to CD23 on B cells. As shown in Figure 2D, FAB was significantly inhibited by sera taken after 4, 12 and 15 months of AIT (means of inhibition: 36%, 26% 47%), respectively. In addition, the capacity of the sera to inhibit allogeneic basophil activation by preventing the binding of allergen to surface‐bound IgE and cross‐linking of FcεRI on the basophils was tested as described in the Data S1. After 4 months, a significant inhibition with a mean value of 48% was observed, which slightly increased to 54% after 1 year. Interestingly, no further increase in BAT‐inhibition (56%) was observed after 15 months (Figure 2E).

To verify that our tests to assess blocking capacity were working optimally, we compared it to the blocking capacity of sera from 8 patients who had been vaccinated with unmodified, alum‐adjuvanted, subcutaneous AIT with GP‐allergen (characterization see Ref. 1, 4, 5 in Data S1). The treatment protocol for the latter had comprised a phase of dose increase during the first 4 months, followed by applications of maintenance doses at monthly intervals. The levels of GP‐specific IgE, IgG, IgG1 and IgG4 in the control sera were assessed in parallel with the sera of the patients treated with allergoid within the same ELISA experiment (Figure S1A). No significant changes in GP‐specific IgE‐levels were observed. GP‐specific IgG, IgG1 and IgG4 subclass antibodies were increased after 4 months, and further increased after 13 months of AIT and reached similar levels as the allergoid‐treated patients.

The FAB inhibition with sera from patients who had undergone AIT with unmodified allergens reached a mean of 40% after 4 months and increased significantly to 86% after 13 months of AIT (Figure S1B). Moreover, inhibition of basophil activation reached a mean of 59% at 4 months and increased to 93% after 13 months of AIT (Figure S1C). Thus, both assays showed similar results within each patient group. However, the IgE‐blocking capacity assessed under the same experimental conditions was significantly higher in sera from patients treated with unmodified allergens after 13 months of AIT compared to patients treated with allergoid after 15 months of AIT (unpaired Student's *t* test; FAB: *P* < .0001, BAT: *P* = .0173).

Thus, preseasonal AIT with formaldehyde‐modified GP‐allergens induced IgG1‐ and IgG4‐antibodies specific for GP‐extract as well as for defined major grass allergens in our patient group, indicating—in line with previous studies^1^, ^2^, ^5^, ^6^—that this allergoid retains immunogenicity and at least part of the original, native Ig epitopes. Interestingly, the production of allergen‐specific IgG4 antibodies started later than the production of IgG1 in several patients and the drop at 12 months was less prominent. This observation may possibly relate to a preferred sequential switching from IgG1 to IgG4 in B cells after repeated vaccination.

Most of the data reported on blocking antibodies induced by AIT derive from studies using vaccines with unmodified allergens. We employed two different biological assays to evaluate the IgE‐blocking activity in sera of allergoid‐treated patients: one involved the inhibition of complex formation between IgE and allergen (FAB) and the other the capacity to inhibit IgE cross‐linking (BAT) (Figure 2D,E). Both assays revealed functional blocking activity in patients’ sera. As the extent of blocking in these inhibition assays depends on the experimental set‐up, we analysed in parallel the blocking activity in sera from patients who had previously been treated with unmodified allergens. Containing similar levels of AIT‐induced allergen‐specific IgG, IgG1 and IgG4 (Figure S1A) and under the very same experimental conditions, the sera of this control group showed significantly higher inhibition, in both assays (Figure S1B,C).

Neither the individual concentration of allergen‐specific IgG antibodies nor the decrease in serum levels during the interruption of vaccination correlated with the individual blocking capacity. For instance, in spite of the general reduction in GP‐specific IgG, IgG1 and IgG4 after 12 months, the IgE‐blocking activity had further increased, suggesting that not total IgG levels are relevant, but rather other properties, such as epitope specificity or affinity of the IgG antibodies. In line, it has been shown for AIT with unmodified allergens that the IgE‐blocking activity of post‐AIT sera does not correlate with the levels allergen‐specific IgG4 but rather with IgE‐blocking capacity.^3^, ^7^ Interestingly, Aasbjerg *et al* 8 have demonstrated that IgE‐blocking was already maximally induced after the first 3 months of AIT with unmodified allergens. In our study, in both, AIT with allergoid or with unmodified allergens, IgE‐blocking capacity was also already present after 4 months, but had not yet reached its maximum level.

In contrast to the treatment with allergoids, the individual data of the BAT‐inhibition assay were more coherent in patients treated with unmodified allergens. We conclude that in a vaccine containing native allergens, most IgE‐epitopes are present and accessible, so that a greater variety of antibodies can be induced which may lead to a more uniform total IgG response and high total blocking capacity. Inversely, the lower blocking capacity observed in sera of patients with allergoid‐treatment is most probably due to the modification of the tertiary structure of the allergens. Studying immune responses to AIT with unmodified birch pollen, we previously found that the majority of IgG4 antibodies recognize similar epitopes on the major birch pollen allergen (Bet v 1) as IgE antibodies of the same patient.^9^ As allergens in allergoids possess modified surface structures, the epitope specificity of the antibodies induced during AIT may be at least partially different from the epitopes of antibodies that have been induced by natural allergens. As some of the IgE‐epitopes on allergens are not accessible in allergoid formulations, blocking IgG antibodies against these epitopes presumably cannot be induced by AIT.

In summary, this study demonstrates for the first time that antibodies induced by AIT with allergoids possess functional IgE‐blocking capacity in vitro. As the ex vivo data on basophil activation induced by GP‐extract revealed a marked reduction during vaccination in these patients (Figure 1B) and the basophil reactivity with anti‐IgE had not changed during the treatment (data not shown), we suggest that the reduction in basophil reactivity is most probably due to the presence of functional blocking antibodies in vivo. It would have been of special interest whether the IgE‐blocking antibodies that can be induced by allergoids suffice to induce tolerance in the patients. However, no data regarding the clinical efficacy of the vaccination with allergoid in our patient group are available and we cannot correlate IgG levels or IgE‐blocking data to the clinical improvement of the patients. For definite statements on the role of blocking antibodies in AIT with allergoids, DBPC studies with higher patient numbers and appropriate clinical evaluation definitely are required.

## CONFLICT OF INTEREST

FH has retrieved fees for organizing education and for attending symposia from ALK, Hørsholm, Denmark and Allergopharma, Reinbeck, Germany. SM has received reimbursement for attending a scientific symposium from Allergopharma. The remaining authors have no conflict of interest to declare.

## References

[1] Calderon MA , Alves B , Jakobson M , Hurwitz B , Sheikh A , Durham S . Allergen injection immunotherapy for seasonal allergic rhinitis (Protocol). Cochran Database Syst Rev. 2007;1: CD001936. 10.1002/14651858.CD001936.pub2 PMC7017974 17253469

[2] Brehler R , Kahlert H , Thum‐Oltmer S . Immunological features and their impact on clinical efficacy and safety, exemplary for the allergoids Allergovit^®^, Acaroid^®^, and a folding variant of the recombinant birch pollen major allergen Bet v 1. Allergo J. 2010;19:477‐484.

[3] Shamji MH , Ljorring C , Francis JN , et al. Functional rather than immunoreactive levels of IgG4 correlate closely with clinical response to grass pollen immunotherapy. Allergy. 2012;67:217‐226. 22077562 10.1111/j.1398-9995.2011.02745.x

[4] Wurtzen PA , Lund G , Lund K , Arvidsson M , Rak S , Ipsen H . A double‐blind placebo‐controlled birch allergy vaccination study II: correlation between inhibition of IgE binding, histamine release and facilitated allergen presentation. Clin Exp Allergy. 2008;38:1290‐1301. 18510696 10.1111/j.1365-2222.2008.03020.x

[5] Özdemir SK , Sin AS , Güloglu D , Ikinciogullari A , Genctürk Z , Misirigil Z . Short‐term pre‐seasonal immunotherapy: is early clinical efficacy related to the basophil response? Int Arch Allergy Immunol. 2014;164:237‐245. 25170594 10.1159/000365628

[6] Keskin Ö , Tuncer A , Adalioglu G , Sekerel BE , Sackesen C , Kalayci O . The effects of grass pollen allergoid immunotherapy on clinical and immunological parameters in children with allergic rhinitis. Pediatr Allergy Immunol. 2006;17:396‐407. 16925684 10.1111/j.1399-3038.2006.00442.x

[7] James LK , Shamji MH , Walker SM , et al. Long‐term tolerance after allergen immunotherapy is accompanied by selective persistence of blocking antibodies. J Allergy Clin Immunol 2011;127:509‐516. e501‐505. 21281875 10.1016/j.jaci.2010.12.1080

[8] Aasbjerg K , Backer V , Lund G , et al. Immunological comparison of allergen immunotherapy tablet treatment and subcutaneous immunotherapy against grass allergy. Clin Exp Allergy 2014;44:417‐428. 24734285 10.1111/cea.12241

[9] Subbarayal B , Schiller D , Mobs C , et al. Kinetics, cross‐reactivity, and specificity of Bet v 1‐specific IgG4 antibodies induced by immunotherapy with birch pollen. Allergy. 2013;68:1377‐1386. 24053565 10.1111/all.12236

