# Supplementary figures and images for: Alum‐adjuvanted allergoids induce functional IgE‐blocking antibodies
Source: Clin Exp Allergy. 2018 Mar 23;48(6):741–4. doi: 10.1111/cea.13120 (PMC6001745; doi:10.1111/cea.13120)

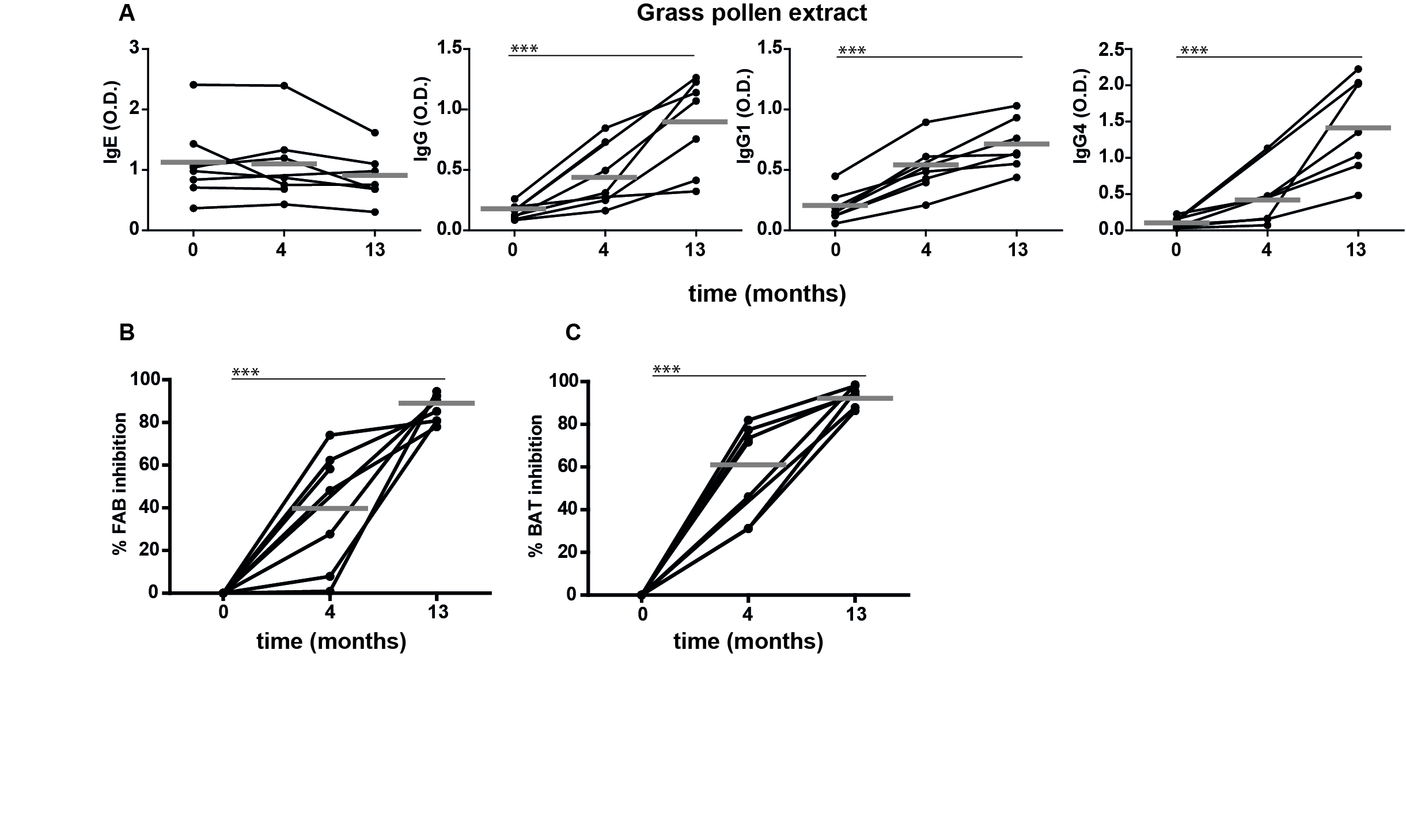

Supplement: Supplementary file 1 [file CEA-48-741-s001.tif]
