# Supplementary material for: Alum‐adjuvanted allergoids induce functional IgE‐blocking antibodies
Source: Clin Exp Allergy. 2018 Mar 23;48(6):741–4. doi: 10.1111/cea.13120 (PMC6001745; doi:10.1111/cea.13120)
Supplement: Supplementary file 2 [file CEA-48-741-s002.docx]

**CEA-2017- 0367 Supplementary Material:**

**Alum-adjuvanted allergoids induce functional IgE-blocking antibodies**

Running head: Allergoids induce IgE-blocking IgG

M. Reithofer^1^, S.L. Böll^1^, C. Kitzmüller^1^, F. Horak^2^, M. Sotoudeh^2^, B. Bohle^1^, B. Jahn-Schmid^1^

^1^Institute of Pathophysiology and Allergy Research, Medical University of Vienna, Vienna, ^2^ Allergiezentrum Wien West, Vienna, Austria

**Corresponding author:**

Beatrice Jahn-Schmid, PhD

Institute of Pathophysiology and Allergy Research

Währinger Gürtel 18-20, AKH 3Q

1090 Wien, Austria

Tel. +43-1-40400 51330

FAX. +43-1-40400 51300

e-mail: Beatrice.jahn-schmid@meduniwien.ac.at

**Patients**

Inclusion criteria for the study were: an age of 18 - 60; a diagnosis of allergy to grass pollen on the basis of typical seasonal symptoms, positive skin prick test and specific IgE. The study was approved by the local ethics committees (No. EK-05/14 and No. 1593/2016) and all patients gave written informed consent.

**Table S1: Characteristics of the study population**

|  |  |  |  |  |  |  | specific IgE (kIU/L) | |
| --- | --- | --- | --- | --- | --- | --- | --- | --- |
| Patient | Age | Sex | Sensitization* | Manifestation** | total IgE (kU/L) | Phl p | Bet v | Der p |
| 1 | 19 | M | gp, bi | RC | 827,0 | >100 | 54,7 |  |
| 2 | 30 | M | gp | RC | 46,7 | 13,2 |  |  |
| 3 | 43 | M | gp, bi | RC | 151,0 | 2,2 | 12,8 |  |
| 4 | 32 | M | gp | RC | 62,0 | 3,5 |  |  |
| 5 | 21 | F | gp, bi | RC | 446,0 | 26,2 | 4,6 |  |
| 6 | 27 | M | gp, bi | RC , Aab | 209,0 | 22,5 | 57,0 |  |
| 7 | 36 | M | gp, bi, hdm | RC | 64,7 | 8,2 | 4,3 | 2,9 |
| 8 | 33 | F | gp,hdm | RC | 52,6 | 5,47 |  | 5,4 |
| 9 | 33 | F | gp | RC | 169 | 11,5 |  |  |
| 10 | 28 | M | gp | RC | 561,0 | 63,7 |  |  |

* gp, grass pollen; bi, birch pollen; hdm, house dust mite;

**RC, rhinoconjunctivitis, Aab, allergic asthma bronchiale

Patients 1, 3 and 6 got SIT with Allergovit against grass pollen and birch pollen in parallel;

Total serum IgE and GP-specific IgE was determined by ImmunoCAP (Thermo Fischer Scientific, Phadia AB, Uppsala, Sweden

The patients were treated with Allergovit® (Allergopharma, Joachim Ganzer AG, Deutschland) in a pre-seasonal regimen (Fig. 1). This allergoid vaccine contains pollen extracts of 6 grass species with about 25µg of Phl p 5 or homologous allergens in the peak dose [reviewed in ref. 2]. As a positive control for our assays, sera from 8 GP-allergic patients (3 female, 5 male; mean age 26.5) from a prior study were analyzed who had undergone AIT with a vaccine containing native GP extract (Alutard SQ 200; ALK) [supplement ref. 1]. Both vaccines have been tested by immunization of mice or rabbits for their immunogenicity. It was shown that the allergoid vaccine Allergovit™ has less [supplement ref. 2, 3] or similar [supplement ref. 4] immunogenic properties than Alutard™, which contains native allergens.

**Methods**

**Basophil activation test (BAT)**

Basophil activation [supplement ref. 5] was assessed in fresh, heparinized blood from allergoid-treated patients. 100µl blood was incubated for 15 min at 37°C with 20 µl of GP extract at final concentrations of 1000, 200, 40, 8, 1.6 ng/ml in HEPES calcium buffer (HCB: 20 mM HEPES, 133 mM NaCl, 5 mM KCl, 7 mM, CaCl_2_, 3,5 mM MgCl_2_, 1 g/l BSA; pH 7.4) supplemented with 2 ng/ml human IL-3 (PeproTech, Rocky Hill, NJ). HCB plus IL-3 served as negative, 1 µg/ml anti-human IgE (Nordic Immunological Laboratories, Maidenhead, UK) or 2 µM fMLP (Sigma-Aldrich) as positive control. After adding 20 µl 20 mM EDTA in PBS, cells were stained with CD63-PE, CD123-FITC and CCR3-APC (all from Biolegend, San Diego, CA), or the respective isotype controls (all from BD Biosciences, San Jose, CA). After lysis of erythrocytes, the percentage of degranulating CD63^+^ cells within the CD123^+^CCR3^+^ basophil population was assessed by flow cytometry.

**Ig ELISA**

The levels of GP-specific IgE, IgG, IgG1, and IgG4 in patients’ sera were determined by ELISA in duplicates. Microtiter plates (Maxisorp, Nunc, Denmark) were coated with 50 μg/ml PBS-extract from GP (Thermo Fischer Scientific, Allergon, Ängelholm, Sweden) or 5µg/ml of the recombinant GP major allergens Phl p 1 or Phl p 5 (Biomay, Vienna, Austria), respectively, in carbonate buffer (pH 9.6) overnight at 4°C. After washing and saturation of unspecific binding, the sera were added at a dilution of 1:5 for IgE, 1:100 for IgG and 1:10 for IgG1 and IgG4 overnight at 4°C. Alkaline phosphatase-conjugated anti-human IgE (BD Biosciences, San Jose, CA) and the substrate *p*-nitrophenyl phosphate (PNPP; Sigma-Aldrich, St. Louis, USA) and mouse anti-human IgG, IgG1 or IgG4 (BD Biosciences) together with horseradish peroxidase–conjugated sheep anti-mouse IgG (GE Healthcare, Bucking­hamshire, UK) and the substrate 2,2'-azinobis [3-ethyl­benzo­t,hiazoline-6-sulfonic acid]-diam­monium salt (ABTS; Sigma-Aldrich, St. Louis, MO) respectively, were used for detection.

**Facilitated allergen binding (FAB)**

To assess inhibitory activity regarding IgE-allergen complex formation in sera of patients, inhibition of FAB to CD23 was determined.The same indicator serum containing more than 100 kU/l of GP-specific IgE was used for all tests. It contained IgE against Phl p 1, Phl p 5, Phl p 4, Phl p 6, Phl p 7, and Phl p 12 as tested by ImmunoCAP ISAC array (Thermo Fischer Scientific). 20 µl indicator serum and 15 µl test serum or an equal volume of medium were pre-incubated with 5 µl of 0.1 µg/ml GP extract for 1 h at 37°C. Then, 1 x 10^5^ cells of a B cell line expressing CD23 in 5 µl medium were added and incubated for 1 h at 4°C. After washing and saturation with 20% human AB serum for 20 min at 4°C, cells were stained with FITC-labeled anti-human IgE (1:10) (KPL, Gaithersburg, MD). After washing, IgE-positive B cells were detected by flow cytometry using a BD FACS Canto II and Diva software (BD Biosciences). 5000 viable cells per sample were acquired. The percentage of IgE-positive cells obtained with pre-AIT sera was normalized to 100%. The percentages of inhibition of IgE/allergen-binding are shown as mean of duplicates.

**Statistics**

Statistical analyses were performed with Student’s t test or Kruskal-Wallis and Dunn’s post hoc test (GraphPad Software Inc., LaJolla, USA). Differences were considered significant if p was ≤0.05.

**Supplemental references:**

1. Ebner C, Siemann U, Bohle B, Willheim W, Wiedermann U, Schenk S, Klotz F, Ebner H, Kraft D, Scheiner O. Immunological changes during specific immuno­therapy of grass pollen allergy: reduced lympho­proliferative responses to allergen and shift from TH2 to TH1 in T-cell clones specific for Phl p 1, a major grass pollen allergen. *Clin Exp Allergy* 1997; 27:1007-1015.

2. Henmar H, Lung G, Lund L., Petersen A, Würtzen PA. Allergenicity, immunogenicity and dose-relationship of three intact allergen vaccines and four allergoid vaccines for subcoutaneous grass pollen immunotherapy. *Clin Exp Immunol* 153:316-23.

3. Lund L, Henmar H, Würtzen PA, Lund G, Hjortskov N, Larsen JN. Comparison of allergenicity and immunogenicity of an intact allergen vaccine and commercially available allergoid products for birch pollen immunotherapy. *Clin Exp Allergy* 2007: 37: 564-571.

4. Weber M, Niespodziana K, Linhart B, Neubauer A, Huber H, Henning R, Valenta R, Focke-Tejkl M. Comparison of the immunogenicity of BM32, a recombinant hypoallergenic B cell epitope-based grass-pollen allergy vaccine with allergen extract-based vaccines. *J Allergy Clin Immunol* 2017 (in press); DOI: <http://dx.doi.org/10.1016/j.jaci.2017.03.048>

5. Hoffmann HJ, Santos AF, Mayorga C, Nopp A, Eberlein B, Ferrer M, Rouzaire P, Ebo DG, Sabato V, Sanz ML, Pecaric-Petkovic L, Patil SU, Hausmann OV, Shreffler WG, Korosec P, Knol EF. The clinical utility of basophil activation testing in diagnosis and monitoring of allergic disease. *Allergy* 2015;70:1393-1405.

**Legend to Figure 1S:** GP-specific Ig-responses and IgE-blocking capacity in patients undergoing AIT with unmodified allergen. IgE, IgG, IgG1 and IgG4 levels were assessed by ELISA. Blocking capacity of sera was determined by blocking the formation of IgE/allergen complexes (FAB assay) (C) or by blocking activation of basophils with GP-extract from an allogeneic GP-allergic individual (BAT assay) (D). Percent inhibition as compared to pre-treatment levels is shown. Kruskal-Wallis and Dunn’s post hoc test; ***p≤0.001). Sera were tested in parallel to sera from allergoid treated patients shown in Figure 2.
